# New Epidemiological, Clinical and Economic Data for Patients With Acromegaly in Bulgaria

**DOI:** 10.3389/fpubh.2020.00147

**Published:** 2020-04-28

**Authors:** Maria Kamusheva, Silvia Vandeva, Konstantin Mitov, Yanitsa Rusenova, Atanaska Elenkova, Sabina Zacharieva, Zornitsa Mitkova, Konstantin Tachkov, Maria Dimitrova, Miglena Doneva, Dimitar Tcharaktchiev, Guenka Petrova

**Affiliations:** ^1^Department of Organization and Economics of Pharmacy, Faculty of Pharmacy, Medical University–Sofia, Sofia, Bulgaria; ^2^Department of Endocrinology, USHATE “Acad. Ivan Penchev”, Medical University–Sofia, Sofia, Bulgaria

**Keywords:** acromegaly, costs, epidemiology, treatment, Bulgaria

## Abstract

**Background:** Acromegaly and its comorbidities affect the patients' quality of life, each healthcare system and the society. This study aimed to evaluate clinical characteristics and treatment patterns and the economic burden of acromegaly.

**Materials and methods:** All patients with acromegaly treated with expensive medicines and regularly followed up at the main expert clinical center for acromegaly in the country were included in this nationwide, retrospective, observational, population-based study. Patient characteristics, treatment patterns, healthcare resource use, and costs were assessed for 1-year period (01.01.2018–31.12.2018). Results were processed through statistical analysis using MedCalc software version 16.4.1.

**Results:** A total of 191 acromegaly patients were observed. Approximately 67% were female, 45.5% were between 41 and 60 years and the mean age at diagnosis was 40.73 years. Surgical treatment was preferred as a first-line therapy among almost 89% of all diagnosed patients. The level of comorbidities was very high as more than 95% suffered from at least one concomitant disease. The most frequent comorbidities were other endocrine and metabolic diseases (96.7%), followed by cardiovascular diseases (70.7%). The most common first-line pharmacotherapy was long-acting somatostatin analogs (SSA) (38%) followed by dual combination SSA + pegvisomant (21%). The total economic burden of acromegaly was estimated to be 2,674,499.90 € in 2018 as the direct costs (medication costs, hospitalization costs covered by the patients and the National Health Insurance Fund) outnumbered indirect costs (loss of productivity due to hospitalization): 2,630,568.58 € vs. 43,931.32 €. The average annual per-patient direct and indirect costs were 14,002.62 €.

**Conclusions:** The current study demonstrates a significant clinical and socio-economic burden of acromegaly in the country. Proper diagnosing and regular follow up of acromegaly patients in a specialized pituitary center ensure appropriate innovative pharmacotherapy with achievement of disease control.

## Introduction

Acromegaly is a rare chronic debilitating disease affecting 2–11 people per million annually. Worldwide, the prevalence of acromegaly is in the range of 28–137 cases/million population (1). According to an epidemiological study carried out in 2010, the estimated prevalence in Bulgaria is around 49 cases/million (2). Currently, the annual number of Bulgarian health insured patients with acromegaly or pituitary gigantism, whose therapy is covered by the National Health Insurance Fund (NHIF), is up to 200 (3).

Growth hormone hypersecretion is related to numerous comorbidities leading to increased mortality due to cardiovascular, cerebrovascular and respiratory diseases (4). In the last years, it became evident that in patients with long follow up cancer displaces cardiovascular diseases as a main cause of death (5–7). However, achievement of disease control restores life expectancy to levels similar to the general population (8, 9). On the other hand, acromegaly has a negative impact on health-related quality of life that barely changes after achievement of remission (10). All these highlight the need for prompt diagnosis, strict control and provision of the most appropriated therapy through close monitoring.

Acromegaly and its comorbidities affect negatively not only the patients' quality of life but also each healthcare system and the society as a whole. An assessment of the real economic and clinical burden of acromegaly is essential for patients and their families, healthcare providers, decision-makers and society. Moreover, the entrance of innovative treatment for acromegaly requires timely and regular pharmaco-economic evaluations. A systematic review performed by the authors of the current manuscript presented the available economic evaluations in the literature (11). In fact, a wide range of studies was identified over a period of 20 years. The studies found in the literature focus more on the cost-effectiveness of the innovative therapies whereas the number of costs of illness studies, especially in some regions such as Central and Eastern European countries (CEEC) region, is scarce. Cost of illness studies or cost analysis describing the economic burden of acromegaly in the CEEC regions are published only for 2 countries: Poland (12, 13) and Bulgaria (14, 15). However, the number of costs of illness studies and other economic evaluations about acromegaly and acromegaly treatments in Bulgaria is still insufficient. Only 2 studies presented as posters at conferences were identified in the literature (14, 15). This enhanced our interest to carry out a more comprehensive study aiming to evaluate clinical characteristics, treatment patterns, and the economic burden of acromegaly.

## Materials and Methods

### Study Design

An observational retrospective study was conducted among acromegaly patients diagnosed, treated and monitored in the reference center for rare endocrine diseases in the country in 2018.

### Setting, Patient Recruitment and Sample Size

All patients treated for acromegaly in were included in the study. The hospital is the only pituitary center in the country supporting a computerized register for patients with acromegaly on SSAs and Pegvisomant treatment, which allowed us to include all patients in the country. The study period was 01.01.2018–31.12.2018.

### Characteristics and Clinical Outcomes

The collected data included demographic characteristics (age, gender); clinical data (acromegaly duration; year of diagnosis, insulin-like growth factor 1 (IGF-1)- and growth hormone (GH)- levels at the first and last hospitalization for 2018, response to applied therapy, remission rate); level of adherence to the prescribed therapy; pharmacotherapy and other therapy: surgery and/or radiotherapy (2018); comorbidity; healthcare resources utilization (hospitalizations per year; medicines utilized); days off work due to acromegaly. Clinical data was gathered from the patients' medical records (data in the electronic database and patients' paper files). Disease control was considered with achievement of normal age-adjusted IGF-1 values.

### Health Economic Outcomes

Direct costs (for medicines, hospitalization) and indirect (days out of work due to illness) costs were calculated on the basis of a bottom-up (micro-costing) approach. Healthcare resources consumed by the acromegaly patients included in the study were identified, measured and valued. Pharmacotherapy monthly costs paid by the National Health Insurance Fund (NHIF) and by the patients were presented separately. They were calculated in two time periods: before the first hospitalization in 2018 and after the last hospitalization in 2018. Hospitalization costs were calculated taking into account the number of hospitalizations per patient per year and the National Framework Contract for payment of medical services signed by the NHIF and the Medical Union, 2018.

Reimbursement levels and the price per unit paid by the NHIF for all included in the Positive Drug List medicines for acromegaly patients (ICD E22.0) were considered. The unit medicines costs were obtained from the Positive Drug List, and for the other resources from the National Health Insurance Fund website and National Statistical Institute reports. All calculated pharmacotherapy costs (monthly cost and annual cost), indirect costs (annual cost) and hospitalization costs per year were presented in euro adjusted for the purchasing power parity (PPP) for 2018 (16). The nominal exchange rate from the European Central Bank was used to convert the national currency (BGN) in euro. The received values in euro were adjusted applying the purchasing power parities based on gross domestic product from the Eurostat website (7, 17, 18). It was calculated that 1 Bulgarian lev (BGN) is equal to 0.5136 PPP-adjusted euros for 2018.

Having the number of hospital days for every patient we calculated the indirect costs [days out of work (absenteeism)] applying the human capital approach formula:

(1) $$\begin{array}{l} \text{Indirect costs =}\frac{\begin{array}{l} Number\;of\;days\;out\;of\;work\times \\ \;average\;monthly\;earnings\;for\;the\;country\;for\;2018 \end{array}}{number\;of\;working\;days\;per\;1\;month\;for\;2018} \end{array}$$

The cost per unit of resource, the time periods for the collection of data and the sources of data for the unit costs are presented in Table 1.

**Table 1:** Evaluated and included in the study resources.

**Direct medical costs**				
**Type of resources**	**Time period**	**Unit costs paid by the NHIF (PPP EUR 2018)**	**Unit costs paid by patient (PPP EUR 2018)**	**Sources of data**
**Pharmacotherapy**				Positive Drug List, Annex 1, 01/2018
Cabergoline 0.5 mg	1 month	60.40 €	0.00 €	
	12 months	724.80 €	0.00 €	
Bromocriptine 2.5 mg	1 month	3.32 €	0.00 €	
Quinagolide	1 month	21.13 €	0.00 €	
Octreotide LAR 20 mg	1 month	523.01 €	195.94 €	
	12 months	6,276.09 €	2,351.34 €	
Octreotide LAR 30 mg	1 month	784.61 €	0.00 €	
	12 months	9,415.27 €	0.00 €	
Pasireotide LAR	1 month	2,780.49 €	0.00 €	
	12 months	33,365.92 €	0.00 €	
Pegvisomant 10 mg	1 month	2,009.46 €	0.46 €	
	12 months	24,113.48 €	5.53 €	
Pegvisomant 15 mg	1 month	3,014.18 €	0.00 €	
	12 months	36,170.19 €	0.00 €	
Pegvisomant 30 mg	1 month	6,028.36 €	0.00 €	
	12 months	72,340.38 €	0.00 €	
**Hospitalization**	12 months	256.80 €	2.98 € per day	National Framework Contract, 2018 (clinical path 80.1); Decree No. 193 of the Council of Ministers, 2012
**Indirect costs**				
**Type of resources**	**Time period**	**Number of working days per month**	**Average monthly earnings (PPP EUR 2018)**	**Sources of data**
Days out of work	12 months	20	555.46 € PPP	National Statistical Institute report, 2018

The total annual direct costs paid by the public fund were found as a sum of the annual pharmacotherapy and hospitalization costs. The total costs from societal perspective presented the sum of all direct medical and indirect costs per year for all acromegaly patients.

### Statistics

A set of statistical methods were applied to describe and assess the correlations among data of interest: descriptive statistics, non-parametric analysis Kruskal-Wallis, Wilcoxon test for paired samples, chi-squared test, and regression analysis. MedCalc statistical software version 16.4.1 for biomedical research was used. Descriptive statistics were applied to present the basic characteristics of the patients: sex, age, birthplace, age of diagnosis, type of therapy. Comparisons between the median values of different patients' groups characteristics were performed by the Kruskal-Wallis test. Wilcoxon test was used to compare paired samples regarding the levels of IGF-1 before and after treatment, whereas the chi-squared test was useful for comparison of distributions and relationships between categorical variables (type of remission, response to therapy, etc.). Regression analysis predicted the outcomes based on historical data regarding the year of diagnosis and the number of patients diagnosed.

### Ethics

All patients with acromegaly were asked to take part in the study. All patients with no exception provided signed written informed consent at their admission authorizing the use of their anonymized (pseudonymized) data for scientific purposes. The local hospital ethics committee approved the study (number of approval is 4/09.08.2019).

## Results

### Demographics

All acromegaly patients (*n* = 191) treated in the University hospital “Acad. Ivan Penchev,” Sofia for the year 2018 were enrolled in the study. Significantly more women were affected as they represent ~67% of the sample (*p* < 0.0001). Patients who live in urban areas are almost 3 times more than those from villages (*p* < 0.0001) which is in accordance with the national statistic data for the proportion of the urban and rural population in the country−1:2.8 The highest was the number of patients between 41 and 60 years of age—almost half of all enrolled (*p* < 0.0001) (Table 2).

**Table 2:** Demographic characteristics of acromegaly patients population in Bulgaria (*n* = 191).

**Category**	**Sub-group**	**Number [distribution in %]**
Age	<40 years	35 [18.3%]
	40 – 60 years	87 [45.5%]
	>60 years	69 [36.1%]
Gender	Male	64 [33.5%]
	Female	127 [66.5%]
Region	Village	40 [20.9%]
	Town/city	151 [79.1%]

### Clinical Characteristics

The mean age at diagnosis for acromegaly was 40.73 years (95% CI 38.901–42.553, SD = 12.7). There was no statistically significant difference between the median age at diagnosis for acromegaly for both genders (*p* = 0.16) and place of birth (city or village) (*p* = 0.5662). 171 patients (89.5%) were subjected to transsphenoidal adenomectomy (TSA) and their mean age was 41.13 years (95% CI: 39.09–43.15), whereas only 18 patients at mean age of 46.33 years (95% CI: 37.85–54.82) were contraindicated for TSA due to concomitant illness or a refuse to be operated. The rest two newly diagnosed patients were referred to the neurosurgical unit for operation.

In our cohort 118 patients have been treated with SSA at some stage of their follow up. At the time of the study full response to the therapy, evaluated by normalization of IGF-1, was achieved in 59 patients (Table 3). All patients on continuous SSA monotherapy with active disease, or patients on combination therapy, including SSA and some other class of drugs, or monotherapy with Pasireotide LAR were considered as partial responders (*n* = 46). Patients on monotherapy with Pegvisomant or combination therapy with Pegvisomant and Cabergoline were considered as non-responders (*n* = 8), as all of them have previously been treated with SSA. In five patients SSA therapy was newly initiated and the therapeutic response could not be evaluated. At some time during the follow up 35 of our patients were treated with Pegvisomant as monotherapy or in combinations with SSA and/or cabergoline.

**Table 3:** Clinical characteristics of acromegaly patients in Bulgaria.

**Category**	**Sub-group**	**Values**
**Clinical data**
Mean age at diagnosis (years) n = 191	Male	39.08 years of age (35.9-42.25)^*^ [SD = 12.71]
	Female	41.58 years of age (39.33-43.82)^*^ [SD = 12.67]
IGF-1 levels^**^ at the first hospitalization for 2018	Male (*n* = 29)	28.7 [16.25-36.95]^†^
	Female (*n* = 55)	29.7 [17.45-37.83]^†^
IGF-1 levels^**^ at the last hospitalization for 2018	Male (*n* = 29)	28.5 [19.3-36.4]^†^
	Female (*n* = 55)	22.5 [14.48-30.40]^†^
GH-levels^***^ at the first hospitalization for 2018	Male (*n* = 28)	6.85 [2.45-10.85]^†^
	Female (*n* = 51)	7 [3.73-18.9]^†^
GH-levels ^***^ at the last hospitalization for 2018	Male (*n* = 28)	4.7 [1.63-7.18]^†^
	Female(*n* = 51)	4.7 [2.65-8.83]^†^
Response to Sandostatin LAR therapy *N* = 118	Non-responders	8 (6.78%)
	Partial responders	46 (38.9%)
	Responders	59 (50.0%)
Remission rate *N* = 191	Active disease	30 (15.7%)
	Disease control	161/191 (84.3%)
Patient reported outcomes^****^		
Medication adherence	Adherence	57 males (89.1%); 120 females (94.5%)
	Non-adherence	7 males (10.9%); 7 females (5.5%)

* *95% CI*.

** *nmol/L (reference value:)*.

*** *mIU/L (reference value:)*.

**** *include information about health-related quality of life (HRQOL), symptoms, function, satisfaction with care or symptoms, adherence to prescribed medications or other therapy, and perceived value of treatment (19)*.

† *median value [25-75P](hypothesis for normal distribution was rejected)*.

The levels of IGF-1 and GH in males were lower at the end of the last compared to the first hospitalization for 2018: 28.7 vs. 28.5 [no statistical significance (*p* = 0.888)] and 6.85 vs. 4.7 [no statistical significance (*p* = 0.189)], respectively. Different observations were found for the female group: 29.7 vs. 22.5 for IGF-1 [no statistical significance (*p* = 0.051)] and 7 vs. 4.7 for GH [statistical significance (*p* = 0.041)]. As a percent of all patients treated with Octreotide LAR more than half of them respond to the prescribed therapy (38.9% were partial responders and 50% were full responders). Correlation analysis showed statistically significant correlation between remission rate and response to Octreotide LAR therapy as more responders to Octreotide LAR achieve full remission: 26 patients are full responders and they are in remission [contingency coefficient = 0.447 (*p* = 0.0194)]. Logically, statistically significant more patients who adhered to the prescribed therapy have achieved full remission in comparison with non-adherent patients: 93.18 vs. 6.82% (*p* = 0.001). Similar results were observed for rate of remission and level of adherence as adherence to therapy logically ensured higher level of response (*p* = 0.0005).

**Figure 1:**
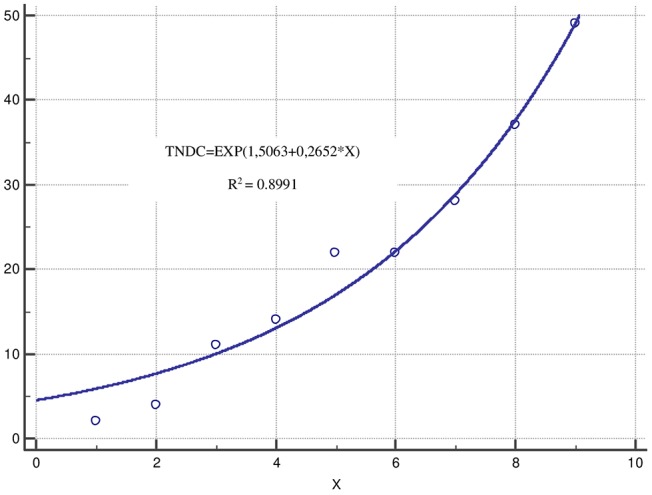
Exponential regression analysis. *TNDC, number of diagnosed patients with acromegaly; X, years presented in intervals each of 5 years for the period 1975–2018.

Interesting results were observed after applying an exponential regression analysis for the variables “number of diagnosed patients with acromegaly” (TNDC) and “year of diagnosis” (X). The regression model has the following analytical equation: TNDC=EXP(1,5063+0,2652^*^X) (Figure 1). It is an adequate model (*p* = 0.0001) with high coefficient of determination (0.8991) and low standard error (2.848) for the period of time (1975–2018). The number of patients diagnosed for this period has been increasing and the number of acromegaly patients for the next 5-year period of time could be prognosed with a high reliability.

### Co-morbidity

Almost 17% of acromegaly patients were diagnosed with at least one concomitant disease as those with more than 2 diseases prevailed−152 patients or 80%. More than 95% of all patients suffered from other endocrine, nutritional, and metabolic diseases (Table 4).

**Table 4:** Concomitant diseases in acromegaly patients in Bulgaria.

**Concomitant diseases**	**Total [%]**	Number [%]^*^ <40 years	Number [%]^*^ 40-60 years	Number [%]^*^ >60 years
No	7 [3.66%]	5 [2.62%]	1 [0.52%]	1 [0.52%]
Yes	184 [96.34%]	30 [15.7%]	86 [45%]	68 [35.6%]
>2 diseases	152 [79.6%]	12 [6.3%]	74 [38.7%]	66 [34.5%]
Only 1 disease	32 [16.8%]	18 [9.4%]	12 [6.3%]	2 [1.04%]

* *Percentages are calculated as the number of patients in each group is divided by the total number of patients (n = 191)*.

**Figure 2:**
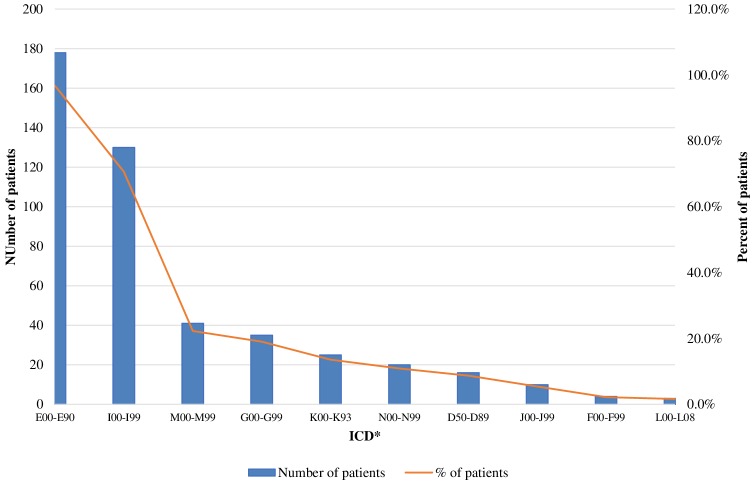
Acromegaly patients with concomitant disease (absolute and relative values). *ICD, International Statistical Classification of disease and related health problems 10th revision (ICD-10); E00-E90, Other endocrine, nutritional and metabolic disease; 100-199, Disease of the circulatory system; M00-M99, Disease of the musculoskeletetal system and connective tissue; G00-G99, Disease of the nervous system; K00-K93, Disease of the digestive system; N00-N99, Disease of the genitourinary system; D50-D58, Disease of the blood and blood-forming organs and certain disorders involving the immune mechanism; J00-J99, Disease of the respiratory system; F00-F99, Mental and behavioral disorders; L00-L08, Disease of the skin and subcutaneous tissue.

The mean number of comorbidities was statistically more significant in women than in men (2.73 vs. 2.08) (*p* = 0.00076). A similar trend was revealed for the different age groups as the number of comorbidities was the highest among patients over 60 years of age [1.371 (<40 years), 2.48 (40–60 years), and 3.13 (>60 years)] (*p* < 0.000001). The patients' settlements did not influence the number of comorbidities: 2.38 in villages vs. 2.55 in cities (*p* = 0.739). The most common concomitant diseases among acromegaly patients were other endocrine and metabolic diseases (ICD E00-E90) (96.7% or 178 patients), followed by cardiovascular (ICD I00-I99) (70.7%) and musculoskeletal disorders [ICD M00-M99 (20, 21)] (22%) (Figure 2).

The patients with other endocrine disease or neurological disease spent statistically significant more days in hospital in comparison with those without these diseases: 8.53 vs. 6.15 days (*p* = 0.012) and 9.51 vs. 8.10 days (*p* = 0.02), respectively.

### Pharmacotherapy and Treatment Patterns

The most prescribed medicines were SSA as almost 39% of all patients were treated with Octreotide LAR in 2018. Pasireotide LAR was applied as part of clinical trials program and only 1.6% of patients were indicated for this therapy. Combination therapy included dual or triple combinations mainly between SSA (Octreotide LAR) and dopamine agonists (Cabergoline) or SSA and Pegvisomant. Combination therapy was prescribed to almost 20% of all patients at first hospitalization and at last 2018 hospitalization.

**Figure 3:**
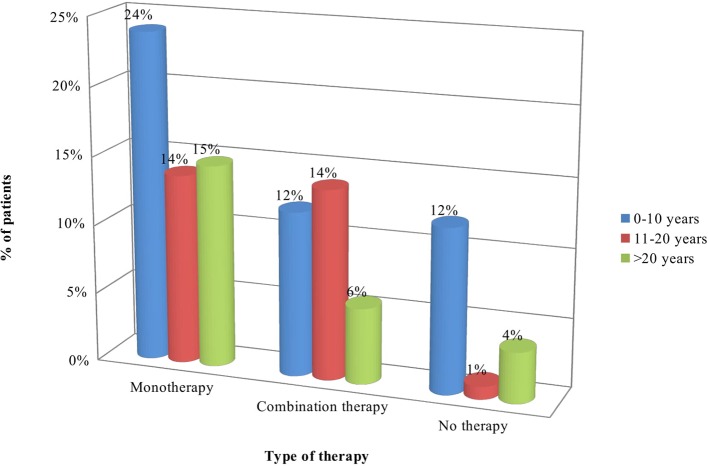
Type of therapy according to disease duration.

Statistically significant difference was observed between distribution of patients according to the type of pharmacotherapy and duration of the main disease: more patients (26) diagnosed with acromegaly 1–10 years ago were on monotherapy in comparison to those diagnosed 11-20 years (13) and > 21 years (13) ago (*p* = 0.0357). Longer disease duration was associated with lower number of patients without therapy (4 patients vs. 15 on monotherapy and 6 on combination therapy) (*p* = 0.0357). Combination therapy was prescribed mainly to patients diagnosed 11–20 years ago (*p* = 0.0357) (Table 5, Figure 3).

**Table 5:** Acromegaly pharmacotherapy in Bulgaria by type of therapy and by INN.

**Category**	**Number [distribution in %]**
**Pharmacotherapy by type at first hospitalization in 2018 (*****n*** **= 191)**	
Monotherapy	88 [46%]
Combination therapy	40 [21%]
No therapy	63 [33%]
Pharmacotherapy by INNs^***^ at first hospitalization in 2018 (***n*** **= 191)**	
DA^*^	10 [5.3%]
SSA^**^	73 [38.2%]
Pegvisomant	2 [1.1%]
SSA+DA	9 [4.7%]
Pegvisomant + SSA	22 [11.5%]
Pegvisomant+DA	6 [3.2%]
SSA+DA+Pegvisomant	3 [1.6%]
Pasireotide LAR	3 [1.6%]
**Pharmacotherapy by type at last hospitalization in 2018 (*****n*** **= 188)**	
Monotherapy	91 [48.4%]
Combination therapy	39 [20.7%]
No therapy	58 [30.9%]
**Pharmacotherapy by INNs at last hospitalization in 2018 (*****n*** **= 188)**	
DA^*^	12 [6.3%]
SSA^**^	74 [39.4%]
Pegvisomant	2 [1.06%]
SSA+DA	9 [4.8%]
Pegvisomant + SSA	21 [11.2%]
Pegvisomant+DA	6 [3.2%]
SSA+DA+Pegvisomant	3 [1.6%]
Pasireotide LAR	3 [1.6%]

* DA, dopamine agonists;

** SSA, somatostatin analogs;

*** *INNs, international non-proprietary names*.

### Healthcare Resources Utilization and Costs

Logically, the costs paid by patients and the NHIF increased by elevating the number of hospitalizations and length of hospital stay (*p* < 0.0001) (Table 6). The length of hospital stay was longer in patients diagnosed with other endocrine disease: 8.53 days [SD = 1.9] vs. 6.15 days [SD = 3.34] as it was statistically significant (*p* = 0.012). Total costs for hospital stay paid by the Fund for all patients with acromegaly for 2018 were ~79 900 euro, whereas the total out-of-pocket payments for hospitalization were 4,726.29 euro (Table 6).

**Table 6:** Healthcare resources use, direct and indirect costs (€PPP).

**Type of costs**	**Patients**	**Number, average**	**Mean costs per patient paid by NHIF (€PPP) [SD]**	**Mean costs per patient paid by patient (€PPP) [SD]**	**Total costs per year (NHIF) (€PPP)**	**Total costs per year (patients) (€PPP)**
**Direct medical costs**						
1. **Medications**						
Monotherapy^*^	88	-	753.28 [830.63]	240.47 [82.74]		
Combination therapy^*^	40	-	3,565.37[1,908.09]	328.94 [127.79]		
**Total annual costs for medications**					**2,275,486.82**	**270,490.67**
2. Hospitalization ^**^ (number per year)						
1 hospitalization	83	5.67 days	256.80	16.69 [5.68]		
2 hospitalizations	96	9.85 days	513.60	29.80 [6.58]		
3 hospitalizations	12	14.58 days	770.40	43.46 [7.67]		
**Total annual costs for hospitalizations**					**79,864.80**	**4,726.29**
**Type of costs**	**Patients**	**Number, average**	**Mean costs per patient per year (**€**PPP) [SD]**		**Total costs per year**	
**Indirect costs**						
1. Lost productivity ^**^(Absenteeism)	191	8.34 days	222.30 [93.36]		**43,931.32**	

* cost per month;

** *cost per year. NHIF, National Heath Insurance Fund; PPP, Purchasing Power Parity; SD, Standard Deviation. Some values are bold in order to highlight their importance*.

Mean monthly medicines costs paid by the NHIF were significantly higher for patients on combination therapy than on monotherapy: 3,565.37 €PPP vs. 753.28 €PPP, respectively (*p* < 0.000001). A similar result was observed for out of pocket monthly payments: 240.47 €PPP for monotherapy vs. 328.94 €PPP for combination therapy (*p* = 0.000001). Total annual reimbursable costs for all acromegaly patients are almost 2,280,000.00 €PPP. Total patients' costs per year reached 270,500.00€PPP.

Median monthly NHIF reimbursement pharmacotherapy costs paid per patient were 784.59 €PPP (95% CI 523–1045.99, SD = 1784.07) while out of pocket pharmacotherapy payments were 195.94 €PPP (95% CI 195.94–319.18, SD = 107.04).

**Figure 4:**
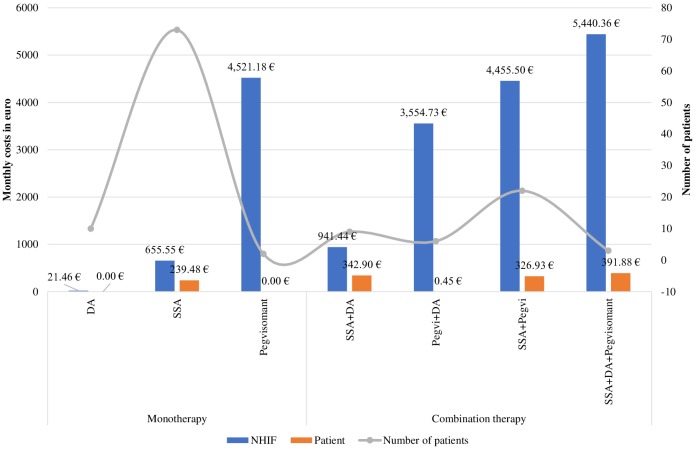
Monthly costs or acromegaly pharmacotherapy for 2018 by therapeutic groups. *DA, dopamine agonists; SSA, somatostain analogs; Pegvi, Pegvisomant.

Pharmacotherapy costs varied in a wide range depending on the type of therapy and prescribed dosage scheme: Octreotide LAR could be prescribed in doses of 20 mg, 30 mg or 40 mg per month given I.M. intragluteally. Pegvisomant is administered by daily subcutaneous injection at three possible doses: 10, 15 or 30 mg per day etc. Therefore, the average costs presented in Figure 4 differ significantly as the highest were the NHIF costs for triple combination (SSA+Pegvisomant+DA) 5,440.36 €PPP, followed by monotherapy with Pegvisomant (4,521.18€PPP) prescribed in higher dose in comparison with combination therapies SSA+Pegvisomant (4,455.50 €PPP) or Pegvisomant+DA (3,554.73 €PPP).

**Figure 5:**
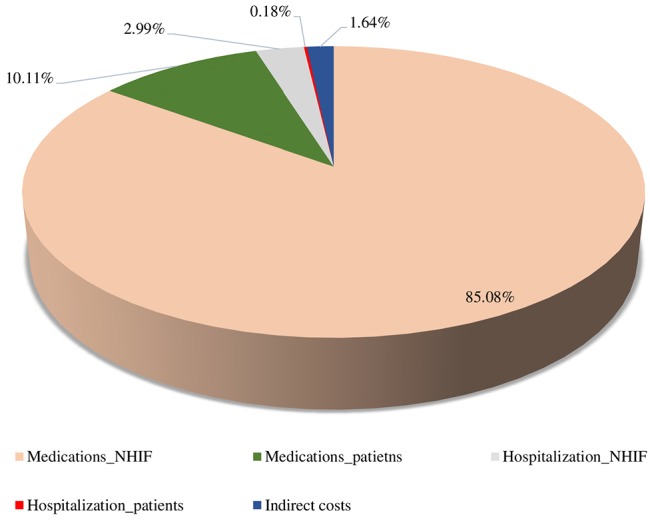
Distribution of direct medical and indirect costs for acromegaly.

Total direct (hospitalization and pharmacotherapy) and indirect costs are presented in Figure 5 as the pharmacotherapy costs covered by the NHIF (85.08%) far outnumber the other costs (14.92%).

## Discussion

The current study presents the demographic and clinical characteristics of acromegaly patients and evaluates the available treatment patterns among the observed group of Bulgarian patients. Moreover, the monthly and annual total costs related to acromegaly were calculated for the Bulgarian reality from three perspectives (society, NHIF, patients). The perspectives were chosen having in consideration the specificity of the Bulgarian health insurance system. The Health Insurance Act from 1998 introduces mandatory health insurance in Bulgaria and gives a legal basis for the establishment of NHIF as an independent public institution. The NHIF reimburses partially or fully medicinal products, medical devices, dietetic foods, foods for special purposes for the treatment of obligatory health insured Bulgarian citizens. A methodological approach has been developed and published for the inclusion of the medicines in the reimbursement lists as specific clinical and economic criteria are included. A strict economic analysis regarding the financial burden of each disease especially of rare diseases is crucial from both NHIF and patients perspective (22).

It was revealed that Bulgarian female patients with acromegaly were almost double than male (127 vs. 64) and those between 41 and 60 years prevailed−45.5%. Some published studies found the same difference in the frequency according to patients' gender (23–25) while in others both genders were affected equally (26–28). We found that the mean age at diagnosis for acromegaly was 40.73 years in Bulgaria which is in accordance with the literature data (between 40 and 50 years of age) (24, 29–31).

The number of patients diagnosed with acromegaly in Bulgaria is increasing following exponential trend established in an analysis over a long period of time: 1975–2018. This might be explained with improved diagnostic tests, widespread use of magnetic resonance imaging (MRI) (32) and increased awareness for this specific group of patients. The exponential regression model could be used for the purposes of prognostic analysis of the expected number of acromegaly patients and related costs for diagnosis, surgery, hospitalization, treatment, and follow up in the near future. Further studies focusing on incidence should be provided so as to clarify this tendency.

Surgical treatment (TSA) was preferred as a first-line therapy in almost 90% of our patients. Similar figures were reported from our nationwide database (2, 33), as well as in numerous national registries (Finland (31), Italy (34), Spain (35), Canada (36), South Korea (37), Germany (38), France (7), Mexico (39). Radiotherapy was performed in 16.7% of all 191 patients, in contrast to the data from our registry, where close to 30% of all patients were irradiated. Similar tendency for reduction of the use of radiotherapy has been observed in several registries (7, 26, 31, 36). It could be explained by the introduction of highly effective medical treatment, such as SSA and Pegvisomant, leading to improved biochemical control. However, in our cohort the number of irradiated patients could be underestimated as the study period is only one year and presumably patients with stable disease control after radiotherapy visit our clinics less frequently.

Pharmacological treatment was applied in 68% of our cohort. This figure could be overestimated in terms of giving a representative picture of the whole country due to the presence of less frequently followed up patients. However, as SSAs and Pegvisomant are prescribed only in our center, our study presents all patients in the country on these medications. Long-acting SSAs was the most prescribed therapy (61.7%), as a mono- or combination therapy, in accordance with the current guidelines in the European region and Bulgaria (40). Monotherapy with SSA led to disease control in 50% (59 out of 118 patients with prescribed SSA at any time of the follow up) of our patients. We assume that it is a real-life picture as it has been a first-line therapy for all patients with persistent moderate or severe disease activity after TSA, or as a primary therapy. Our remission rates are in agreement with the results from a recent meta-analysis, showing 55% normalization of IGF-1 under long-acting SSAs (41).

Dual combination SSA + pegvisomant is the second most prescribed therapy in the observed group (10.9% of all patients). Pegvisomant was used extremely in a combination therapy – 93.7% of all patients under this treatment, in part due to cost-effective issues. Of note, an increase in the proportion of combination therapy has been observed in a recent update of the ACROSTUDY (42). The overall remission rates under treatment with Pegvisomant, as mono- or combination therapy at any point during the follow up was 82.8% which is a bit higher from other real-life data (43). It could be explained by a beneficial effect of close monitoring and prompt titration of doses in a centralized manner in a specialized pituitary center. This speculation can be valid for the overall remission rates of our cohort of 82.3%.

Our patients suffered mostly from other endocrine and metabolic diseases (ICD E00-E90) (96.7% or 178 patients) such as diabetes mellitus, disorders of thyroid gland, hypopituitarism, hyperprolactinemia etc., followed by cardiovascular (ICD I00-I99) (70.7%) (arterial hypertension, arrhythmias, cardiomyopathy etc.) and musculoskeletal disorders (ICD M00-M99) (22%) (arthropathy, osteoarthritis, osteoporosis etc.). In a recent study Maione et al. review data from national registries on comorbidities, the most prevalent being arterial hypertension (11–54%), type 2 diabetes mellitus (12–40%), osteoarthropathy (20–69%), and pituitary hormone deficiency (8–68%) (7). Normalization of GH and IGF-1 are essential for control of comorbidities and subsequently reduction in mortality rates, which become similar to the general population in the recent reports (5–7, 34). The socio-economic burden of comorbidites is a complex issue and deserves a more detailed study.

The economic evaluation of acromegaly was performed among 191 patients and it is the first common study that gathered information for all diagnosed and treated in 2018 Bulgarian patients. The economic burden of acromegaly was estimated to be 2,674,499.90 € in 2018 as the annual per patient costs are approximately 14,002.62 € taking into account direct and indirect costs. Another Bulgarian study among smaller group of acromegaly patients calculated that total direct costs per patient per year are around 14,800.00€ which is a little bit higher probably due to the sample size (15). A Swedish study calculated higher annual costs equal to 6,328,000.00€ in 2013 whereas the annual per patient costs were lower – 12,000.00€ (43). Similarly to this Swedish study, we found that direct costs outnumber the indirect costs. However, we should remark that the indirect costs calculated in the current study are associated only with the days out of work due to hospitalization which underestimate the real indirect costs related to acromegaly.

Interestingly, the NHIF costs outweighed almost nine times the out of pocket payments which indicates adequate assurance of Bulgarian acromegaly patients with financial access to innovative therapies. The NHIF pays annually almost 2,270,000.00 € for acromegaly pharmacotherapy—a result similar with the results of a previously conducted Bulgarian study in which the top-down approach for costs calculation was applied (14).

To the best of our knowledge, this is the first study carried out in Bulgaria and focusing on the clinical and socio-economic burden of acromegaly. It could serve as an initial step for further national and regional longitudinal incidence-based cost of illness studies for better understanding the real impact of acromegaly from various perspectives: the societal, patients', their families' and the National healthcare insurance fund's.

A strong limitation of our study is the short period of observation (only 1 year) taking into account narrow range of costs: only for medicines, hospital stay and temporary loss of productivity. Broadening the calculated costs taking into consideration presenteeism, informal care, concomitant diseases treatment, and direct non-medical costs such as transportation due to required health care visits, surgery costs as well as presenting more detailed analysis of the effectiveness on the basis of real-world data could bring more value to the research and could better inform the stakeholders.

Moreover, this study stresses the importance of further combined economic and clinical evaluations for other rare diseases in Bulgaria. Thus, enough economic and clinical information could be gathered for the purposes of making healthcare decisions based on valuable scientific evidence.

## Conclusions

The current study demonstrates a significant clinical and socio-economic burden of acromegaly in Bulgaria. Early diagnosis and regular follow-up of acromegaly patients in Bulgaria ensure adequate access to specialized medical care and appropriate pharmacotherapy. Bulgarian acromegaly patients have been provided with financial access to the latest innovative therapies.

## Data Availability Statement

The datasets generated for this study are available on request to the corresponding author.

## Ethics Statement

The studies involving human participants were reviewed and approved by Local Ethics Committee in USHATE Acad. Ivan Penchev, Medical University-Sofia, Bulgaria (number of approval is 4/09.08.2019). The patients/participants provided their written informed consent to participate in this study.

## Author Contributions

All the authors have provided valuable contributions to the manuscript. MK and SV carried out the research and drafted the manuscript. MK and YR entered the patients' data in a database. KM, MK, and MDo performed the statistical analysis. AE, SZ, ZM, MDi, DT, KT and GP participated in the study design and reviewed the paper. All authors read and approved the final manuscript.

## Conflict of Interest

The authors declare that the research was conducted in the absence of any commercial or financial relationships that could be construed as a potential conflict of interest.
